# Supplementary material for: A Global Screen for Assembly State Changes of the Mitotic Proteome by SEC-SWATH-MS
Source: Cell Syst. 2020 Feb 26;10(2):133–155.e6. doi: 10.1016/j.cels.2020.01.001 (PMC7042714; doi:10.1016/j.cels.2020.01.001)

Q9HDC9 | APMAP_HUMAN | APMAP C20orf3 UNQ1869/PRO4305
Monomer MW [kDa]: 46.48 Monomer expected elution fraction: 47

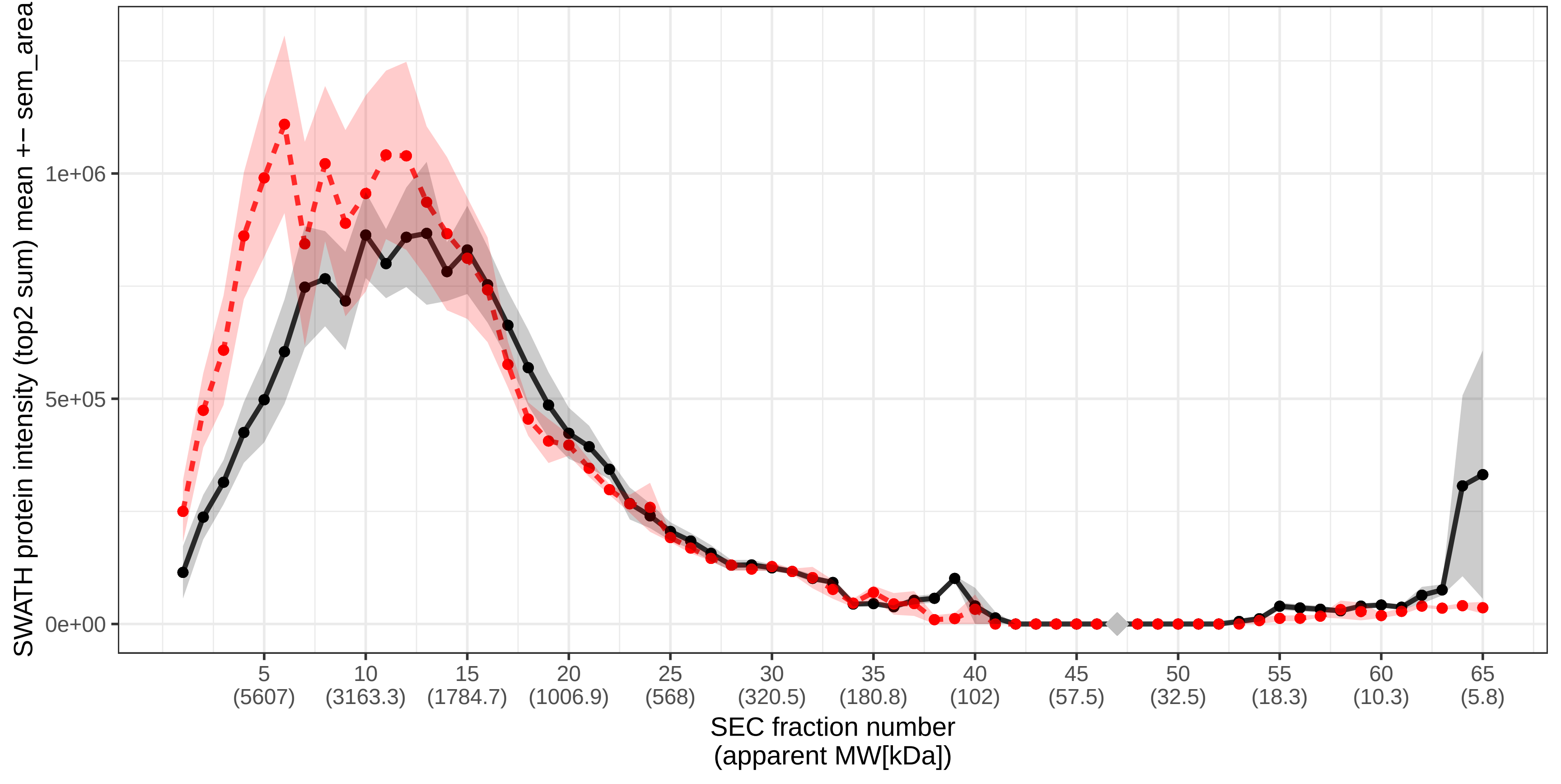

Supplement: Data S1. SEC-SWATH-MS Protein Chromatograms, Related to Figure 1 [file mmc6.zip › SECchrom_Q9HDC9_APMAP_HUMAN_APMAP_C20orf3_UNQ1869_PRO4305.pdf]
